# Evolution of Dengue Disease and Entomological Monitoring in Santa Cruz, Bolivia 2002 – 2008

**DOI:** 10.1371/journal.pone.0118337

**Published:** 2015-02-23

**Authors:** Philippe Brémond, Yelin Roca, Simone Frédérique Brenière, Annie Walter, Zaira Barja-Simon, Roberto Torres Fernández, Jorge Vargas

**Affiliations:** 1 INTERTRYP (CIRAD- IRD, Interactions hôte-vecteur-parasite dans les maladies dues aux Trypanosomatidés), Institut de Recherche pour le Développement (IRD), Montpellier, France; 2 Centro Nacional de Enfermedades Tropicales, Santa Cruz, Bolivia; 3 Servicio Departamental de Salud, Santa Cruz, Bolivia; Georgia State University, UNITED STATES

## Abstract

**Background:**

In the context of a rapid increase of dengue cases in the Americas, a monitoring system based on systematic serological control (IgM) of patients consulting for suspected dengue was developed in Bolivia at the end of the 1990s. In the most affected city of Santa Cruz, this system was complemented by an entomological surveillance program based on periodical search for immature stages of *Aedes aegypti* in dwelling water-holding containers. Here, we analyze these data and describe dengue patterns over 6 years (2002–2008), highlighting the spatial distribution of patients and vectors.

**Methodology /Principal Findings:**

Data mining concerned six annual epidemic cycles (2002–2008), with continuous serological and clinical results and entomological data from 16 surveys, examined at the scales of 36 urban areas and four concentric areas covering the entire city. Annual incidence varied from 0.28‰ to 0.95‰; overall incidence was higher in women and adults, and dengue dynamics followed successive periods of high (January–June) and low (July–December) transmission. Lower numbers of cases from the city center to the periphery were observed, poorly related to the more homogeneous and permanent distribution of *A. aegypti*. "Plant pots" were a major vector source in the city center, and "Tires" and "Odds and ends" beyond the second ring of the city.

**Conclusions/Significance:**

Over the years, the increasing trend of dengue cases has been highlighted as well as its widespread distribution over the entire city, but an underestimation of the number of cases is strongly suspected. Contrary to popular belief, the city center appears more affected than the periphery, and dengue is not particularly related to waste. Interestingly, the clinical diagnosis of dengue by physicians improved over the years, whatever the gender, age and residential area of suspected cases.

## Introduction

Dengue is currently considered the most common arthropod-borne viral infection in the world, emerging or already spreading in most tropical and subtropical countries, with 50–100 million infections annually and over half of the world population at risk (WHO; see <http://www.who.int/topics/dengue/>. The disease is caused by any of four closely related viruses of the genus *Flavivirus*, DEN-1, DEN-2, DEN-3, and DEN-4, and transmitted through the bite of *Aedes* genus mosquitoes, mainly *A. aegypti* and, to a lesser extent, *A. albopictus*. In Latin America dengue has become a public health challenge since the 1980s, with a dramatic increase in the number of cases during the 2000–2010 decade [1], mostly associated with fast-growing urban centers. Today, 60% of worldwide reported dengue cases are observed in the Americas [2].

In Bolivia, dengue fever was mentioned for the first time in 1931 [3], but well-documented cases during the 1990s appeared with the isolation of DEN-1 and DEN-2 viruses [4,5,6], followed by DEN-3 at the beginning of the 2000s [3]. Supported by the Rockefeller Foundation, the *A. aegypti* eradication was completed in 1948, but the mosquito reappeared during the 1980s [7] and quickly spread to all tropical parts of the country. *A. aegypti*, the only known dengue vector in Bolivia, has since been commonly reported from the Amazon basin to an altitude close to 2500 m, i.e., in the seven departments of this region except Potosi and Oruro. The Andean part of the country has remained unaffected by dengue, whereas cases are regularly reported in the Bolivian Orient, mostly in the Santa Cruz department. According to the statistics of the Bolivian Ministry of Health and Sports (see <http://www.sns.gob.bo/>, during the 2008–2009 epidemic, 49% of the reported dengue cases in the country originated from the city of Santa Cruz.

At the end of the 1990s, the Bolivian Ministry of Health and Sports and its specialized research institute in Santa Cruz city, the Centro Nacional de Enfermedades Tropicales (CENETROP) and the Servicio Departamental de Salud (SEDES) developed a monitoring system to follow up dengue outbreaks at a national level. This system established mandatory reporting of suspected dengue cases through epidemiological reporting forms, and a systematic sampling of blood for IgM serological control, centralized at the CENETROP, whatever the patients’ region of residence and whether patients consulted in private or public institutions. Moreover, in Santa Cruz city, *A. aegypti* larvae and pupae were actively sought in water-holding containers in two or three visits a year in dwellings.

Because the rapid changes observed in dengue epidemiology in Latin America during the 2000s are still poorly documented [1], it appeared helpful to draw the pattern of the disease in the most alarming endemic area in Bolivia: Santa Cruz city. A first attempt to identify the high-risk areas for dengue in Santa Cruz was made using the 2003–2007 serological data of the surveillance system [8]. The authors reported an increasing trend of dengue cases over the years and a wide distribution in the city, somewhat lower at the periphery. However, the incidence of dengue cases was only compared between two wide areas (central and peripheral); mapping of the reported cases did not take into account population density and only 79% of the data were included. No link was established with the available entomological data.

In the current work, the analysis was extended to six epidemic cycles (2002–2008 period) and linked to the data from the vector surveillance system. The intra- and interannual dynamics of various dengue key parameters were studied, as well as spatial distributions over time at two scales corresponding to 36 urban areas and four major concentric areas covering the entire city.

## Materials and Methods

### Spatial and administrative organization of Santa Cruz city

The city of Santa Cruz was founded at the end of the 16^th^ century and is the most populated city in Bolivia. It is located in the lowlands of the eastern part of the country and today covers about 380 km^2^, with an estimated population of over two million people in 2012. The city has grown by extending ring roads around the city (up to nine, clearly delineated from the first to the fourth one and irregular beyond) and radial avenues (about 30, from the center to the periphery).

The basic units of the main administrative city division, the UVs (*Unidad de vivienda*) and ETs (*Equipamiento*) areas that are included between two rings and two radial avenues could not be used in the present work because 27% of the patient data had an inaccurate address, and upper subdivisions were needed.

However, the upper subdivisions of the city, (i) the *barrio* (neighborhood, with about 350 in the city), with not well defined limits, (ii) the *distritos municipales* (13 municipal districts), which do not cover the entire city, and (iii) the four *redes de salud*, that do not properly represent the historical extension of the city, were not usable for the study.

### Definition of new spatial divisions in Santa Cruz for the current analysis

**Fig 1:**
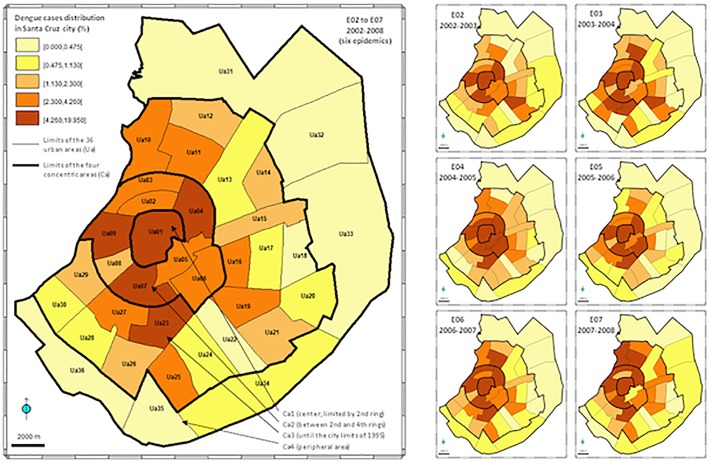
Distribution of dengue cases in Santa Cruz city. Left: overall dengue cases 2002–2008 (six epidemic cycles); right: annual dengue cases (October–September) from E02 to E07. The five quantile classes are similar for all maps and have been defined from overall data. Ua01–Ua36 = urban areas; C1–C4 = concentric areas.

The present city division levels leading to unsatisfactory spatial positioning of the recorded patient cases prompted us to compose two new divisions to improve the spatial exploration of the data. Hence, we divided the city into 36 urban areas (Ua), called Ua01–Ua36 (Fig. 1), which allowed localizing 93% of the patients and all entomological data (already referenced at the UV scale). These Uas grouped a total of 494 UV-like areas including the 446 UV–ET areas referenced by the City Council and 48 additional areas created to cover the whole city and to distinguish the major nonresidential areas (universities, hospitals, customs building, large firms, bus and railway stations, airports, prison, settling ponds, cemeteries, green areas, and the San Aurelio agricultural area included within the city). The limits of the Uas were defined so as to (i) follow the historical development of the city from its center to the periphery and (ii) take into account the lower population densities in the periphery, by including a higher number of UV-like elements within the most external Ua.

A higher-level division of the city into four major concentric areas, named Ca1–Ca4 from the center to the periphery (Fig. 1), was defined for additional analyses. The concentric area Ca1 (8.3 km^2^) corresponds to the most central urban area Ua01, limited by the second ring created during the 1950s. Ca2 (44.0 km^2^) includes nine Uas of similar size (average: 5.5 km^2^ ± 1.5) and is located between the limit of Ca1 and the fourth ring, created during the 1980s. Ca3 (160.7 km^2^) includes 20 larger Uas (average: 7.7 km^2^ ± 2.0) and is externally bounded by the approximate 1995 city limits (close to the eighth and ninth rings). Ca4 (163.5 km^2^) includes the six widest peripheral Uas (average: 27.3 km^2^ ± 15.0) and is extended to the city’s 2005 administrative boundary.

### Parameter acquisition for analyses

We analyzed the monitoring system data using two sources available from the CENETROP and the SEDES, the national patient data set established after the mandatory notification of suspected dengue cases beginning in 2001, and the departmental vector data set established from the entomological surveys conducted since 1998. The “Consejo de Dirección del CENETROP and SEDES” approved this retrospective study. We selected the most reliable data, i.e., 2002–2008 for the analysis that corresponds to six annual epidemiological cycles, starting in October and ending in September of the following year, and defined according to the transmission dynamics (see Results).

Patient data set

The patient data set comprised over 10,000 suspected dengue cases among the inhabitants of Santa Cruz who had freely consulted at public health centers, hospitals, clinics, and private offices. The patients were informed through an individual standardized registration sheet established by the surveillance program, whose data were anonymously used in our study after recoding the name and surname column with numbers. Civil registration data (age, sex, residential address), clinical symptoms recorded according to six categories (fever, rash, hemorrhages, muscular rigidity, jaundice, and respiratory failure), and date of symptom appearance were collected. These data were complemented at the CENETROP with the results obtained after the serodiagnosis applied to all suspected patients, using a MAC-ELISA assay (immunoglobulin M (IgM)-based test) standardized in the laboratory.

The association of the six main symptoms with the dengue fever was examined, comparing their frequencies between unconfirmed and confirmed dengue cases. The date of symptom onset for each patient was used to explore the weekly and monthly dynamics of dengue. Gender and two age classes, children (0–14 years old) and adults (≥ 15 years old), were taken into account for the subsequent analyses. The annual and cumulative incidence over the total population, per sex and per age class were calculated according to the results of the 2001 census in Santa Cruz city, using the demographic data (population size, sex ratio, age distribution) and the annual growth rates estimated by the INE (Instituto Nacional de Estadistica: National Institute for Statistics; <http://www.ine.gob.bo). In the present study, consulting patients suspected of having dengue are the suspected cases, patients with positive IgM serology are the dengue cases, patients with negative or doubtful IgM results are the unconfirmed cases, and the dengue cases / suspected cases ratio is the IgM confirmation rate. According to the laboratory Guidance and Diagnostic of the CDC center (http://www.cdc.gov/dengue/clinicallab/laboratory.html Testing), the MAC-ELISA IgM detection assay has become a routine dengue diagnosis test of approximately a 90% sensitivity and 98% specificity. Even though detection of the virus either by viral culture or PCR remains the definite confirmation test for dengue, it was not possible to be applied in a national scale in Bolivia by the surveillance program.

Entomological data set

The entomological data set included over 130,000 water-holding containers inspected in the search for *A. aegypti* larvae and pupae, mainly in the peridomestic areas (patios) of the dwellings, two to three times a year. For each survey, 20–207 UVs were sampled (average: 124 ± 64 of the 387 officially registered UVs); within each UV, four to 21 residential blocks were visited and, after agreement from the inhabitants, six to 65 dwellings were sampled. During the 16 campaigns conducted over the 2002–2008 period, 31–380 containers per UV were examined and classified according to 11 SEDES categories (tanks, barrels, wells, canals, plant pots, tires, etc.); the latter information was available for the large majority of the positive containers (N = 1500, 94.7%) and for 45,112 negative containers (37.6%). For all campaigns, the following data were available per UV: number of positive and negative residential blocks, dwellings, and containers. However, the information on whether each container belonged to a given dwelling, or whether each dwelling belonged to a given residential block was not available.

The entomological data were considered monthly for the six previously defined epidemic cycles to allow for a comparison with the patient data set. Seasonal analysis was performed according to the rainy (October–April) and dry (May–September) seasons defined from the 1961–1990 meteorological data at the intra-city Trompillo airport (see: <http://www.tutiempo.net/clima/>). The 11 container types were grouped into four main categories: "Tires" (used or not), "Plant pots" (plants and flowers in vases and pots, pot bases, small clay containers), "Odds and ends" (small and other disposable containers, waste), and "Others" (tanks on the roof of the house or on the ground, cisterns, barrels, casks, tubs, wells, canals, trees, and rocks with natural holes).

### Population density, residential indexes and spatial dengue incidence-like

It appeared impossible to estimate spatial dengue incidence in Santa Cruz city, however the city was broken down because (i) the data of the INE 2001 census were available at the level of census areas (*zonas censales*: 262 including about 4,000 inhabitants each) with no link to the corresponding UV–ET and (ii) this census did not cover the whole city: of the 355 inhabited UV–ETs (among the 446 referred by the prefecture), 77 (21.7%) were excluded and 20 (5.6%) were partially surveyed.

Hence, in order to estimate a “dengue incidence-like” at the Ua scale (urban area), we used a residential index (RI) instead of population density values. First, on the basis of 2006 Google Earth images of each UV-like area, the surface area of the residential part was estimated (total area composed of dwellings and peridomestic areas), and the Ua RI was the ratio of the sum of the residential surface areas of the UV-like areas by the total surface area of the Ua; some buildings were assigned based on information requested from SEDES and CENETROP personnel who had very good knowledge of the city. The incidence-like of dengue cases per Ua was then expressed by the number of dengue cases in a Ua / Ua-RI ratio.

### Mapping and statistical analyses

All patients were localized through their address at the Ua scale (and consequently at the Ca scale). The dwellings where entomological surveys were done, already localized in the UV partitions, were easily brought to the Ua and Ca scales. The patients and dwellings attributed data were linked to a geographical information system through the SavGIS software developed by the IRD (Marc Souris: see <http://www.savgis.org>), first used, based on a UV–ET shapefile provided by the prefecture of Santa Cruz, to draw the polygons corresponding to the 494 UV-like areas, the 36 urban areas, and the four concentric areas in specific layers. Maps of the Ua distribution of dengue cases and dwelling infestation rates by *A. aegypti* immature stages were designed for each epidemic cycle and for the overall 2002–2008 data. Additional thematic maps were drawn to highlight the dynamics of dengue during the low transmission periods and the evolving clinical diagnosis indicated by changes in the IgM confirmation rates.

To compare proportions in categories among two or more samples, the chi-square test was applied. The categories explored were sex, age class, patient symptoms, case location, epidemic cycles, and annual transmission periods. Similarly, the vector infestation of dwellings and containers were compared among the epidemic cycles, the four concentric areas, and the type of containers. Correlations (applying a linear regression model) between the distance of each Ua to the central Ua01 (centroid to centroid) and the number of dengue cases, the dengue incidences-like, and the *A. aegypti* container infestation rates per Ua were tested using the R software with F statistics.

## Results

### Six-year dengue surveillance program in Santa Cruz

The passive surveillance of dengue in Santa Cruz city monitored 10,236 suspected patients for whom IgM antibodies to dengue were sought. Results of six dengue epidemics E02 (2002–2003) to E07 (2007–2008) were analyzed.

Reported dengue cases

Table 1 summarizes the results for E02–E07 dengue epidemics. Of the annual estimated population in the city, the average proportion of suspected cases was 1.3‰ ± 0.7 and the average estimated incidence was 0.5‰ ± 0.3. The cumulative incidence of dengue cases increased significantly between the three first epidemics (2002–2005) and the last three (2005–2008) (1.3‰ vs. 1.7‰, respectively, χ^2^ = 52.3, d.f. = 1, *P* < 0.01). However, annual variations were observed, and E06 presented the highest estimated incidence (0.95‰), the others ranging from 0.28‰ to 0.61‰. Annual variations were similar for both gender and age classes. Women showed a higher cumulative incidence (2002–2008) than men (3.2‰ vs. 2.9‰, χ^2^ = 15.1, d.f. = 1, *P* < 0.01). Interestingly, when compared to the overall population (M/F sex ratio = 0.95), women appeared over-represented among the dengue cases (M/F = 0.84, χ^2^ = 15.0, d.f. = 1, *P* < 0.01), but not among the suspected cases (M/F = 0.93, χ^2^ = 0.5, d.f. = 1, *P* = 0.46). Children showed a cumulative incidence lower than adults (1.8‰ vs. 3.7‰, χ^2^ = 348.8, d.f. = 1, *P* < 0.01). Of the overall population, children suspected of harboring dengue were under-represented (26.5% vs. 36.9%, χ^2^ = 460.1, d.f. = 1, P < 0.01) as were children with dengue (22.7% vs. 36.9%, χ^2^ = 364.9, d.f. = 1, *P* < 0.01).

**Table 1:** Distribution of suspected and dengue cases and dengue incidence among gender and age classes in Santa Cruz city from 2002 to 2008.

		Epidemic cycle					
Variable		E02	E03	E04	E05	E06	E07
Overall estimated population^a^		1,213,295	1,264,514	1,316,176	1,368,450	1,421,576	1,475,368
Overall suspected cases (‰)		2.44	1.12	0.65	0.72	1.92	0.87
No. of suspected cases^b^							
	Overall	2,963	1,423	860	980	2,728	1,282
	Female	1,518	769	440	469	1,409	683
	Male	1,440	654	419	511	1,319	599
	0–14 years old	831	378	192	266	637	321
	15–99 years old	2,000	989	652	687	2,036	933
No. of dengue cases (IgM positives)							
	Overall	737	597	366	380	1,351	671
	Female	409	333	203	196	725	363
	Male	327	264	163	184	626	308
	0–14 years old	135	154	72	83	290	176
	15–99 years old	583	430	288	286	1,029	487
Estimated incidence (‰ inhabitants)							
	Overall	0.61	0.47	0.28	0.28	0.95	0.45
	Female	0.66	0.51	0.30	0.28	0.99	0.48
	Male	0.55	0.43	0.25	0.28	0.90	0.43
	0–14 years old	0.30	0.33	0.15	0.16	0.55	0.32
	15–99 years old	0.76	0.54	0.35	0.33	1.15	0.52

^a^From 2001 INE census (*N* = 1,162,949 in 2001) adjusted by annual growth rates of 4.33%, 4.22%, 4.09%, 3.97%, 3.88% and 3.78%, respectively, from E02 (2003 population data) to E07 (2008 data).

^b^There were six missing data for gender and 314 for age.

The symptom occurrences for dengue and unconfirmed cases are presented in Table 2. Almost all suspected patients had fever. Although a significant difference was recorded between the groups with positive and negative IgM (χ^2^ = 15.0, d.f. = 1, *P* < 0.01), this symptom is obviously not specific to dengue, but it should warn of a possible case of dengue (odds ratio (OR) = 2.04). Rash was the second most common clinical symptom (45.8% within the suspected cases), more frequent in patients with dengue (57.1%) than in unconfirmed cases (37.7%, χ^2^ = 281.4, d.f. = 1, *P* < 0.01). Presence of hemorrhage at the time of consultation was observed in 23.4% of the patients and this clinical symptom was also significantly related to dengue cases (27.1% vs. 20.7%, χ^2^ = 40.0, d.f. = 1, *P* < 0.01). According to the odds ratios, dengue cases were more likely than unconfirmed cases to have rash and hemorrhages (OR = 2.2 and 1.4, respectively). The three remaining clinical symptoms (muscular rigidity, jaundice, and respiratory failure) were very rarely observed (< 5% within the suspected cases), and none of them was positively associated with dengue cases.

**Table 2:** Clinical symptoms reported for dengue cases and unconfirmed cases in Santa Cruz city from 2002 to 2008.

			% of patients with presence or absence of clinical symtoms (No. of patients)				
Clinical symptoms		No. of patients with clinical information	Overall (suspected cases)	IgM + (dengue cases)	IgM - (unconfirmed cases)	*P*-value	Odds ratio
Fever		9,957					
	Present		98.3 (9,788)	99.9 (3,557)	97.9 (6,231)	< 0.01	2.04
	Absent		1.7 (169)	1.0 (37)	2.1 (132)		
Rash		7,636					
	Present		45.8 (3,501)	57.1 (1,828)	37.7 (1,673)	< 0.01	2.20
	Absent		54.2 (4,135)	42.9 (1,373)	62.3 (2,762)		
Hemorrhages		7,015					
	Present		23.4 (1,641)	27.1 (800)	20.7 (841)	< 0.01	1.43
	Absent		76.6 (5,374)	72.9 (2,147)	79.3 (3,227)		
Muscular rigidity		6,564					
	Present		4.7 (309)	4.7 (132)	4.7 (177)	> 0.05	1.02
	Absent		95.3 (6,255)	95.3 (2,648)	95.3 (3,607)		
Jaundice		6,554					
	Present		4.3 (281)	2.9 (80)	5.3 (201)	< 0.01	0.53
	Absent		95.7 (6,273)	97.1 (2,691)	94.7 (3,582)		
Respiratory insufficiency		6,601					
	Present		4.4 (288)	2.9 (82)	5.4 (206)	< 0.01	0.53
	Absent		95.6 (6,313)	97.1 (2,703)	94.6 (3,610)		

Spatial and temporal patterns of dengue

Fig. 1 presents the distribution of dengue cases within the 36 urban areas (Ua01–Ua36) included in the four major concentric areas (Ca1–Ca4) defined in Santa Cruz city, for the overall 2002–2008 period and for each of the six epidemic cycles E02–E07. The overall spatial distribution clearly shows that the number of cases decreases from the center to the periphery, with 57.2% of the dengue cases located within Ca1 and Ca2, together accounting for only 13.9% (52.3 km^2^) of the total estimated urban area. The annual distribution patterns are roughly unchanged from one epidemic cycle to the other, confirming the concentration of dengue cases in the central areas Ca1 and Ca2 (65.0% during E02 to 51.4% during E07). According to the estimated surface areas of the four concentric areas Ca1–Ca4, the cumulative density (2002–2008) was 61.3, 38.6, 9.6, and 0.6 dengue cases per square kilometer from the center toward the periphery. Significant negative correlations were observed between the number of dengue cases and the dengue incidence-like per Ua and the distance to the city center (R = −0.73, *P* < 0.01; R = −0.51, *P* < 0.01, respectively).

**Fig 2:**
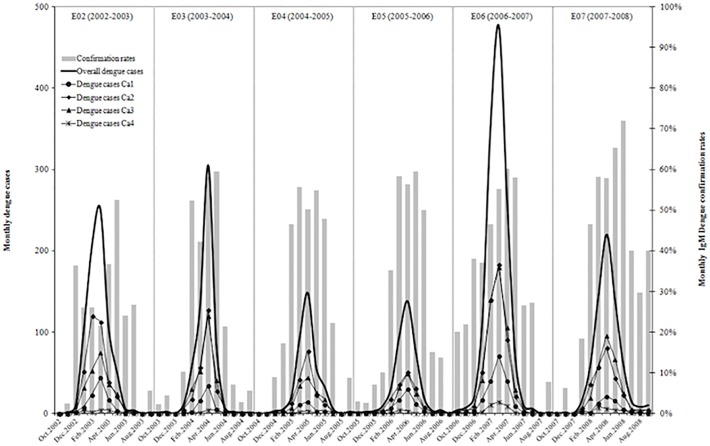
Monthly number of dengue cases and confirmation rates in Santa Cruz city. Dengue cases and confirmation rates were calculated monthly for the entire city and within each concentric area (Ca1–Ca4) for the six epidemics together (E02–E07). The confirmation rate was expressed as a percentage: dengue cases (IgM positive) / suspected cases. Patients missing data on the onset of the symptoms (36) or location (240) were deleted from the analysis.

Fig. 2 shows the monthly dengue cases for the six epidemics (2002–2008), together with the confirmation rates for the 10,075 suspected cases for which the date of symptom appearance was known. Each epidemic cycle was divided into three periods: early (October–December), central (January–June), and late (July–September). For all six cycles, these periods of 3, 6, and 3 months correspond to 5.8%, 90.0%, and 4.1%, respectively, of the suspected cases and to 1.2%, 97.2%, and 1.6% of the dengue cases. Considering the annual variations within the E02–E07 cycles, we observed 77.7%–94.3% of the annual suspected cases and 95.1%–98.8% of the annual dengue cases during the central high-transmission period. Dengue cases were reported every month during the central periods, whereas during a few months of the early and late low-transmission periods, no dengue cases were confirmed.

**Fig 3:**
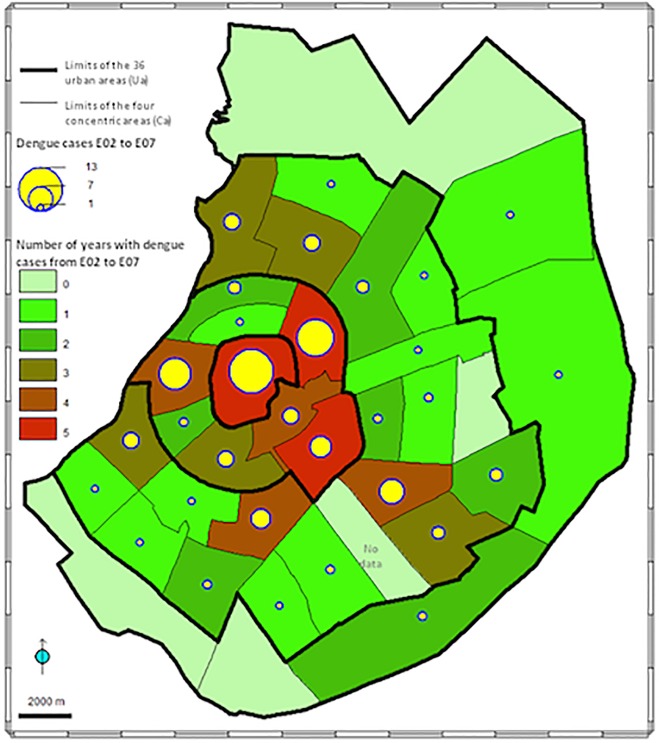
Distribution of dengue cases during the low-transmission periods in Santa Cruz city. The data reported during early (October–December) plus late (July–September) low-transmission periods are presented for the 35 inhabited Uas and for the six epidemics together (E02–E07). The circles are proportional to the number of dengue cases. The Uas are colored according to the number of years with at least one dengue case (varied from 0 to 5).

Examining the monthly spatial distribution of dengue cases in Ca1 to Ca4 (Fig. 2) for the 3,826 positive patients whose residential area was known, a synchronization of the low- and high-transmission periods was observed in the four concentric areas. The spatial distribution of the 105 dengue cases reported during the low-transmission periods (Fig. 3) shows that the patients’ residential areas were dispersed in 88.6% of the urban areas (31/35), each of them presenting 1–5 years of positivity (out of 6) and one to 13 dengue cases for all cycles. In Ca1 and Ca2, the most central areas, these dengue cases were distributed throughout all urban areas (9/9; overall dengue cases: 53), while in the widest peripheral areas (Ca3 and Ca4) these cases were reported in 84.6% of urban areas (22/26; overall dengue cases: 52).

Performance of dengue clinical diagnosis in Santa Cruz

Calculating the dengue confirmation rates for the six cycles (2002 to 2008) from the data presented in Table 1, dengue infection was confirmed for 40.1% of the consulting patients during this period, ranging annually from 24.9% (E02) to 52.3% (E07). Women showed a higher confirmation rate than men (42.2% vs. 37.9%, χ^2^ = 19.4, d.f. = 1, *P* < 0.01), and children a lower rate than patients over 15 years old (34.7% vs. 42.5%, χ^2^ = 49.5, d.f. = 1, *P* < 0.01). Considering the transmission periods (Table 3), the diagnosis appeared more reliable during the high-transmission periods (January–June, 26.2%–56.1%) than during the low ones (3.5%–24.2%), with the overall 2002–2008 data (also see Fig. 2) indicating a significant difference (43.6% vs. 11.4%, χ^2^ = 387.8, d.f. = 1, *P* < 0.01). Moreover, the annual confirmation rates examined at the level of the concentric areas (Table 3) generally tended to decrease from the city center to the periphery, the overall data showing a significant decrease from Ca1 to Ca4 (48.1%, 42.5%, 37.4%, 28.9%, respectively, χ2 = 67.6, d.f. = 3, *P* <0.01).

**Table 3:** Temporal and spatial variations in dengue confirmation rates during the six epidemics 2002–2008 in Santa Cruz city.

	% of confirmation rates (number of suspected dengue cases)					
	E02	E03	E04	E05	E06	E07
Transmission period^a^						
Low (July-Dec.)	9.2 (152)	3.5 (199)	7.5 (147)	6.0 (215)	24.2 (153)	23.7 (139)
High (Jan.-June)	26.2 (2,764)	49.0 (1,204)	50.4 (705)	49.0 (749)	50.9 (2,510)	56.1 (1,138)
Concentric area^b^						
Ca1 (central)	32.2 (311)	50.4 (115)	43.2 (81)	61.2 (116)	58.7 (312)	50.0 (126)
Ca2 (to the 4th ring)	28.2 (1,244)	44.2 (547)	46.2 (381)	43.4 (355)	52.1 (985)	54.6 (476)
Ca3 (to 1995 limits)	20.6 (1,108)	40.9 (618)	39.8 (327)	30.8 (400)	46.0 (1,154)	53.5 (529)
Ca4 (to periphery)	17.6 (85)	22.9 (48)	19.2 (26)	25.0 (32)	37.3 (102)	43.4 (53)

^a^There were 161 undated data

^b^There were 705 unlocalized data.

**Fig 4:**
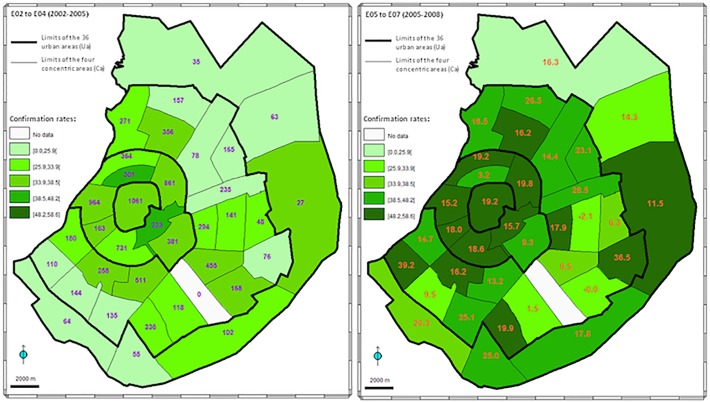
Distribution of confirmation rates of dengue cases in Santa Cruz city. The intensity of the green color of each urban unit (Ua) increases according to a scale of the confirmation rate of dengue cases expressed in percent: left: combined data from the first three epidemics E02–E04 (2002–2005), right: combined data from the last three epidemics E05–E07 (2005–2008). The cumulative number of suspected cases are noted in each urban area for the six epidemics together (E02–E07) in the left picture. The differences of the confirmation rates between the three first epidemics and the last three per Ua, expressed as a percentage, are presented in the right picture.

A clear increase of the confirmation rates was observed between the first three epidemics (E02–E04: 32.4%) and the last three (E05–E07: 48.1%, χ2 = 263.5, d.f. = 1, *P* < 0.01) (Fig. 4), observed during both low- and high-transmission periods (+9.9% and +16.2%, respectively); this increase occurred in most of the Uas ranging from 0.5% to 39.2% for both males and females in the whole population (+15.5% in women; +16.0% in men) and both age classes (+19.1% in children; +13.6% in adults).

### Six-year *A. aegypti* entomological surveillance in Santa Cruz

*Dwelling and container* A. aegypti *larvae and/or pupae infestation rates*

From October 2002 to September 2008, the period corresponding to the six epidemic cycles studied, 16 entomological surveillance surveys by the SEDES / CENETROP were performed in Santa Cruz city (2, 3, 3, 2, 2, and 4, respectively for E02–E07). A total of 131,398 containers were inspected for *A. aegypti* larvae and/or pupae in 35,151 dwellings. The results, summarized in Table 4, show that the dwelling infestation rates ranged from 5.5% to 12.4% (overall: 8.3%) and the container infestation rates from 1.6% to 6.3% (overall: 3.1%), with major values during E04, E05, and E07 in both cases. The comparison of the first three epidemics (E02–E04) with the last three (E05–E07) showed a significant increase of both rates, varying from 7.1% to 10.8% for dwellings (χ^2^ = 142.8, d.f. = 1, *P* < 0.01) and from 2.3% to 5.2% for containers (χ^2^ = 690.5, d.f. = 1, *P* < 0.01).

**Table 4:** Dwelling and container infestations with *Aedes aegypti* in Santa Cruz city.

		Epidemic cycle					
		E02	E03	E04	E05	E06	E07
Dwellings							
	Inspected	7,757	9,022	6,823	4,158	1,219	6,172
	Positives (%)	5.5	6.2	10.1	12.4	7.0	10.6
Containers with water							
	Inspected	35,196	37,299	24,973	11,339	3,765	18,826
	Positives (%)	1.6	2.0	3.8	6.3	3.4	4.8

The dwelling infestation rate was significantly higher during the 7 months of the rainy season (October–April: 8.9%) than during the beginning of the dry season (May–June: 7.5%, χ^2^ = 20.5, d.f. = 1, *P* < 0.01). The container infestation rate did not differ between these periods (3.0% vs. 3.2%, χ^2^ = 3.3, d.f. = 1, *P* = 0.07). No data were available for the last months of the dry season (July–September).

*Spatial distribution of dwellings infested by* A. aegypti *larvae and pupae*

**Fig 5:**
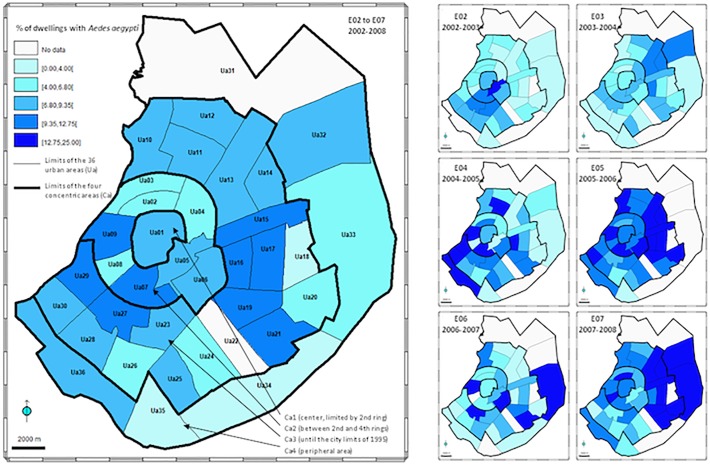
Distribution of dwellings positive for *A. aegypti* larval and pupae stages in Santa Cruz city. Percentage of dwelling infestation by *A. aegypti* larvae and/or pupae per Ua (34 Uas have data). Left: for the 6 epidemics. Right: for each epidemic (October–September). The five quantile classes are similar for all maps and have been defined from overall data (*N* = 189).

The overall and annual distributions of dwelling infestation rates within the 34 urban areas with data (Fig. 5) show a permanent and heterogeneous spread of *A. aegypti* in the city, with annual topologies roughly unchanged from one cycle to another. A significant negative correlation (R = −0.41, *P* = 0.015) was observed between the dwelling infestation rates per Ua and the distances of the Ua to the city center; however, when the Ua whose distance to the center was greater than 9 km were deleted from the analysis, no significant correlation was obtained (R = −0.07, *P* = 0.772). Moreover, similar dwelling infestation rates appeared within Ca1, Ca2, Ca3, and Ca4 (7.8%, 8.3%, 8.5%, and 6.2%, respectively; χ^2^ = 6.0, d.f. = 3, *P* = 0.11). The increase of the infestation rates observed between the first three epidemic cycles (E02–E04) and the last three (E05–E07) at the city level (see above) was also significant in all concentric areas: Ca1 (6.8%–9.8%, X2 = 7.3, d.f. = 1, *P* < 0.01), Ca2 (7.1%–10.9%, χ2 = 45.7, d.f. = 1, *P* < 0.01), Ca3 (7.2%–11.0%, χ2 = 83.4, d.f. = 1, *P* < 0.01), and Ca4 (4.6%–9.8%, χ2 = 7.9, d.f. = 1, *P* < 0.01).

*Types of containers infested by* A. aegypti *larvae and pupae*

Category information on both negative and positive containers was available for 35.5% of the inspected water-holding containers (*N* = 46,612) and considered for the analysis according to the four broad classes (Tires, Plant pots, Odds and ends, and Others (water storage, canals, trees, and rocks)) defined from the 11 types identified by the SEDES (see Material and methods).

**Fig 6:**
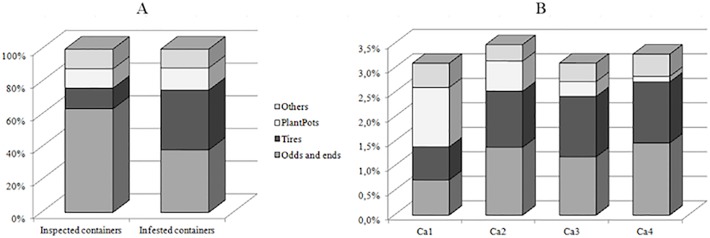
Distribution of inspected and *A. aegypti*-infested container categories in Santa Cruz city. A) Proportions of container categories inspected (potential breeding sites with water) and infested with *A. aegypti* larvae and/or pupae during the six epidemics together. B) Probability of sampling an *A. aegypti*-infested container of each category within each of the four concentric areas Ca1–Ca4; this probability (Pp) is the probability of sampling a category of container × the probability of the container category being positive, expressed as a percentage.

The proportions of these four main categories are presented in Fig. 6A for the overall inspected containers and for those infested with *A. aegypti* larvae and/or pupae. Tires showed the highest positivity rate (9.3%) but accounted for only 12.7% of the overall inspected containers. Plant pots and Others showed similar low positivity rates (3.7% and 3.1%, respectively) and similar low relative proportions (11.8% and 12.1% of overall inspected containers); within the Others category, the highest rate (7.3%) was observed for trees and rocks, uncommonly visited in the city (0.1% of overall containers), whereas the lowest rate (0.1%) was observed for canals (3.1%). The Odds and ends category showed the lowest positivity rate (1.9%) among the four classes of containers, but was the most common one (63.4% of overall inspected containers) and thus appeared as the most represented type within the infested containers (38.3%), together with Tires (36.7%), *versus* 13.5% for Plant pots and 11.5% for Others (Fig. 6A). According to the frequency and the infestation rate for each inspected category, the probability of sampling an infested container in the city was 1.2% for both Tires and Odds and ends, and 0.4% for both Plant pots and Others.

*Spatial distribution of* A. aegypti *container categories*

Of the 46,612 inspected water holding containers classified into four categories, 1.9% were located in Ca4 (the least inspected area) and 4.8% in Ca1 (the smallest area), whereas 30.0% were located in Ca2 and 63.3% in Ca3. The overall infestation rates were similar in the four concentric areas (3.1%–3.5%, χ^2^ = 4.5, d.f. = 3, *P* = 0.21), as observed at the dwelling level, but the relative abundance of the different categories of inspected containers and their infestation rates varied between these areas. In order to evaluate the spatial contribution of each container category to *A. aegypti* larval development, the probability (Pp) of sampling a positive container belonging to each category, in each area, was calculated and is shown in Fig. 6B. The four container categories are breeding sites for the vector in the four concentric areas. Plant pots appeared as the container category with the greatest infestation (Pp = 1.2%) in the center of the city (Ca1), with a decreasing probability of being sampled toward the periphery (0.6%, 0.3%, and 0.1% from Ca2 to Ca3 and Ca4). All three concentric areas beyond Ca1 were characterized by the large number of containers in the Odds and ends (Pp ranging from 1.2% to 1.5%) and Tires (1.1%–1.2%) categories. The category with the fewest containers in the four concentric areas was Others (0.3%–0.5%). Taking into account the density of infested containers per square kilometer, the categories involved in the development of *A. aegypti* were mainly Odds and Ends (4.4 infested containers / km^2^) and Tires (3.6) in Ca2, Plant pots in Ca1 (3.2), and Tires (2.0) and Odds and Ends (1.9) in Ca3.

## Discussion

In the context of a strong increase of dengue cases in the Americas since the 1990s, the surveillance system developed in Bolivia mostly concerns data from the department of Santa Cruz, and especially the city of Santa Cruz, where dengue transmission has been favored by rapid urbanization. So far, the data accumulated since the 2000s from the epidemiological fact sheets and the serological diagnosis, as well as from the entomological surveys, have been only partially analyzed. No detailed analysis has been done because the data need to be completely restructured, with special emphasis on the localization of suspected and confirmed dengue cases. Moreover, the lack of homogenization in the chronological data sets and their dispersion in different institutions prevented a long-term overview of the epidemiological and entomological evolution of dengue in Santa Cruz city. Our study was undertaken so as to provide general and spatialized information on both the disease and the vector at the scale of the city.

### Possible underestimation of dengue in Santa Cruz city

Our analysis of dengue data collected during six epidemiological cycles from 2002 to 2008 in Santa Cruz city involved over 10,000 suspected cases, from which over 4,000 were confirmed by IgM serology. The average annual incidence in Santa Cruz city (0.5‰) appeared lower than what was previously estimated in the Andean subregion including Bolivia (1.0‰) and in the Southern cone countries (1.6‰) during the 2000–2007 period, as well as in Brazil (∼ 2.2‰) during a longer period (1986–2008) [1,9]. It is likely that dengue incidence in Santa Cruz city would be considerably higher, due to the expected strong concentration of the disease mostly in the urbanized areas. Indeed, the consultations for suspicion of dengue in Santa Cruz city concerned only 0.9–2.4‰ of the population per epidemic, while in French Guiana, for example, an average of over 100 cases per week were clinically suspected during the years 2008–2009, around 20–30‰ of the overall population annually [10]. The low consultation level in Santa Cruz city could be explained by the very quick growth of the city, from about 700,000 inhabitants in 1993 to over one million in 2001 and 2.7 million in 2012, which makes more difficult to update the resources needed to support dengue surveillance. Moreover, there is still a broad lack of understanding of the disease by the urban population, approximately 25% migrants, most of them coming from nonendemic regions of Bolivia. Part of the population also prefers the traditional health system and does not use the institutional monitoring system.

Additionally, a recruitment bias stems from the patients who do not participate in the monitoring system because they are diagnosed as infected by the *Mayaro* virus, although its clinical pattern is close to that of dengue fever [11]. The agent, *Alphavirus*, is mainly transmitted by mosquitoes of the genus *Haemagogus* in humid forests of tropical South America, but this vector has never been reported in the city (Le Goff, personal communication). The confusion between the two arboviruses is highly probable.

### Distribution of dengue cases in Santa Cruz city

Various studies on the recent evolution of dengue in South America indicate similar or slightly higher incidence in women than in men and lower but increasing incidence in children than in adults [1,9,12]. Our results fit these observations. One explanation for the higher cumulative incidence in women than in men could be their enhanced exposition to infected mosquitoes because they are more frequently at the bedside of a sick family member. The lower cumulative incidence in children than in persons over 15 years of age suggests a dengue pattern with still low acquired immunity in the overall population, corresponding to a recent increasing epidemic situation that is quite different from the endemic situation currently observed in Southeast Asia, where the four dengue viruses circulate permanently and where the adult population has acquired a strong herd immunity, dengue remaining mostly a childhood disease [13].

The increase in the number of dengue cases from the 1980s until the current period in Latin America was generally highlighted in various endemic countries [1,2,14]. As expected, a significant increase of cumulative incidence was observed in Santa Cruz city over the study period, whatever the gender or age class (data not shown). However, the annual incidence varied, decreasing from 2002–2003 to 2005–2006, followed by a major rise in 2006–2007 and by a second decrease in 2007–2008. These fluctuations are quite similar to those observed in Brazil [9], possibly due to common regional climatic conditions.

Over the 2002–2008 study period, the reported dengue cases were distributed throughout the city but tended to decrease from the center to the periphery, as did the dengue incidence-like, which is an indirect measure of the actual incidence (see Materials and Methods). In fact, the decrease of dengue from the center to the periphery may not be linked to population density, which decreases toward the periphery. An alternative explanation to the variations in spatial dengue incidence could be a stronger concentration of *A. aegypti* breeding sites in the city center, characterized by a higher density of constructions (whatever their use), than in the periphery, as suggested by the estimation of built land occupation (100%, 95%, 65%, and 22% from Ca1 to Ca4).

Seasonality of dengue transmission

The monthly distribution of dengue cases in Santa Cruz city over the six annual cycles indicates very significant seasonal variations. Like the distribution described in Brazil (San Martin et al., 2010), a 6-month high-transmission period (January–June: 97.2% of the overall dengue cases) occurs, surrounded by two 3-month low-transmission periods (October–December: 1.2%; July–September: 1.6%). As expected, the annual transmission profile, with a major concentration of dengue cases during March–April (58.2%) and their extreme rareness during October–November (0.5%), followed the kinetics of rainfall in the city with a 2- to 3- month shift, January being the month with maximum precipitation (mean, 147.6 mm over the study) and August with the minimum (25.8 mm).

Interestingly, a perfect spatial synchronicity of the monthly number of dengue cases was observed between the concentric areas, and these patterns remain unchanged even if a weekly scale was considered (data not shown). This observation supports the hypothesis of a multiple origin of dengue expansion during each epidemic.

The appearance of dengue in various places of the city at the same time should be related to the persistence of cases during the low-transmission periods in almost all urban areas and the widespread distribution of the vector throughout the year. Moreover, the viruses can survive within *A. aegypti* eggs, as already described in Santa Cruz city [15]. Such a widespread and maintained distribution of both vectors and dengue viruses should contribute to a renewed transmission in all parts of the city at the beginning of the rainy season, followed by a quick reemergence of the disease.

*Widespread distribution of* A. aegypti *larvae and pupae in Santa Cruz*

The permanent widespread distribution of *A. aegypti* larvae and pupae, shown by similar dwelling infestation rates in the concentric areas, indicates a lack of correlation with the heterogeneous distribution of dengue cases; however, the study did not involve quantitative data (container counts per dwelling, larvae and pupae counts per container), which could have explained the decrease of dengue incidence-like from the city center to the periphery.

Here, the analysis of the main inspected container categories and their specific infestation rates is more relevant. The results suggest a main *A. aegypty* breeding site in Plant pots within the second ring of the city, although this category is not the most heavily infested. Thus, contrary to what is often believed in Santa Cruz city, well-kept gardens located in the center represent an entomological risk that is almost as important as the risk from, in the other areas, domestic and industrial waste, identified and frequently targeted as the main urban vector source. In South America, the role played by plants, either growing in pots with a water-filled base or kept in vases, was recently highlighted in extradomiciliary areas such as cemeteries [16,17] and in dwellings, in domestic [18] and peridomestic areas [19].

Beyond the second ring of the city, the Odds and ends category (the most numerous type of container among all those inspected) and Tires appear as the major breeding sites of the vector. The impact of used tires on the larval production of *A. aegypti* [20] is well known throughout Latin America [21,22], as is the role played by waste accumulation [23], but in Santa Cruz, as in Brazil [24] and Mexico [25], many other places were found to be breeding sites, non-useful discarded containers such as cans and bottles, for example, more than waste. This observation reveals that Santa Cruz inhabitants tend to keep and store various categories of containers in their yard, including used tires.

A surprising observation is the low number of containers for water storage (cisterns, tanks, barrels, casks, drums, etc.) in Santa Cruz. They are commonly used in Latin America to compensate for the lack of water supply services [26] and frequently observed in the rural areas of Bolivia. In Santa Cruz city, these storage containers represent less than one-tenth of overall inspected and infested containers. With one of the lowest infestation rates (4%), these container categories should not be considered to play a major role in the transmission of dengue infection in Santa Cruz city.

Thus, the regular and efficient public services regarding both trash disposal and water supply appear to limit the spread of *A. aegypti*, whereas the cultivation of ornamental plants and the population’s stockpiling habits appear to favor the development of the vector within the related types of containers. The other potential breeding sites such as tree holes and canals play a marginal role in the city.

### Dengue diagnosis

Among the six principal clinical symptoms included in the epidemiological fact sheet of the patients with dengue fever suspicion, fever (98%) and rash (45.8%) were associated with the disease, as already established [27], while jaundice and respiratory failure were unusual (< 5%) and not associated with dengue. Interestingly, the IgM confirmation rate that we estimated over epidemics and space can be equated to an indicator of medical skills regarding clinical diagnosis of dengue. Considering the overall data, this rate is low (40.1%), reflecting a diagnosis problem due to the atypical symptomatology of dengue and to a confusion with other diseases such as influenza and Mayaro. The confirmation rate is significantly higher in adults than in children (+7.8%), probably because of confusion with other childhood diseases, and higher in women than in men (+4.3%), probably due to a better identification of the disease by the housewives mostly in charge of healthcare within the family and thus able to bring more relevant information to the medical services. As expected, the dengue diagnosis problem is more pronounced during the low-transmission periods (confirmation rate, 11.4%) than during the epidemic peaks (43.6%). One hopeful result is that of the improvement of dengue diagnosis over time, indicated by the significant increase of the IgM confirmation rates from 2002–2005 to 2005–2008 (+15.7%); it concerned the entire consulting population in most of the urban areas (83% with an increase over 9%), which indicates an enhanced identification of the disease at its initial stage whatever the patients’ gender, age, or place of residence.

### What is the best monitoring system for dengue in Santa Cruz city?

The spatial mining of serological and entomological data for the first time made it possible to describe an overall pattern of dengue infection in the largest urbanized area of eastern Bolivian, currently one of the fastest-growing cities in the world. This pattern is characterized by widespread transmission of the viruses (mainly DEN-2 and DEN-3 until 2008) by a unique vector species, *A. aegypti*, with concentration of dengue cases over 6 months. The vector breeding sites are mainly those linked to the cultivation of plants in the city center and to used tires and unused stored items (more than waste). Thus, the frequent popular belief about dengue transmission, involving improper waste management in the peridomicile areas by poorly educated immigrants living mostly beyond the fourth ring, should become a more realistic perception of an epidemiological situation regarding all urbanized areas of the city and "clean" containers as well as domestic and industrial waste.

This work can now offer clues to increasing the knowledge on the dynamics of epidemics and a better coverage of dengue cases.

The current data from the entomological surveillance of *A. aegypti* in Santa Cruz city are limited to the records of presence and absence of larvae plus pupae in the breeding sites, while quantitative results counting at least pupae would be more informative. Indeed, counting *A. aegypti* pupae is a good indicator to estimate the size of the future mosquito populations [28,29]. This improvement could also take into account the original analysis of environmental factors using the Premise Condition Index, established from several house conditions such as tidiness and shade [30,31].

The geographical sampling of *A. aegypti* could also be improved so as to (i) better cover the recently urbanized areas located in the city periphery and (ii) explore residential and nonresidential areas. Indeed, extradomiciliary transmission of dengue is still poorly documented in South America [17] and entomological surveys extended to the areas with major public buildings, schools, markets, cemeteries, parks, bus and railway stations, airports, etc., could provide new useful information. All the entomological sampling could be better distributed over the year, with extensive surveying of breeding sites at the beginning of the rainy season, which could lead to better target preventive insecticide campaigns [32].

Since the infected female mosquito populations determine the epidemic thresholds of dengue infection [33], indoor and outdoor captures of adult *A. aegypti* could also be undertaken [34], followed by the assessment of the vector infection rates through the detection of dengue viruses using real-time PCR, already available at the CENETROP. Applying this method to pools of captured female mosquitoes would provide relevant information on the transmission risks for local scale prevention.

The data from the serological monitoring of dengue did not allow a full description of the spatial distribution of the disease at the finest scale of the UV, used for the entomological surveys, because many addresses of the consulting patients were inaccurate or incomplete. Consequently, we built a higher-level division in 36 wide urban areas, but the UV level, with about 500 units covering an average of 4 km^2^ each for close to 3 million inhabitants remains a suitable scale, and greater care in reporting patient localization data in the epidemiological fact sheet should lead to enhanced detection of the areas to be selected for priority control measures.

On the other hand, the apparently weak herd immunity suggested by the higher incidence in adults than in children could be better assessed by systematically including in the serological analyses the dosage of specific dengue IgG, so that the process of immunization could be followed in particular groups such as recent immigrants or long-term residents, to better target the populations at risk. In addition, because of the frequent confusion between dengue and Mayaro infections in Santa Cruz city (see above), and because *Mayaro* virus, whose epidemiology is currently changing [35], can be experimentally transmitted by *A. aegypti* [36], both diseases should be distinguished through serological analyses applied to all suspected cases of dengue and *Mayaro*, so as to make the serological monitoring system more complete and reliable.

Finally, a few low-cost changes in the dengue monitoring system, based on quantitative sampling of pupa and adult vectors extended in time and space, as well as on precise localization of suspected patients, should easily increase the knowledge of dengue eco-epidemiology in Santa Cruz city, achieving more efficient control of epidemics.

## Supporting Information

S1 Dataset Patient dataset of suspected dengue cases during 2002–2008 in Santa Cruz city, Bolivia. (XLS)
